# Effects of wearing myopia glasses on eye movement and scleral blood supply

**DOI:** 10.3892/mi.2024.179

**Published:** 2024-07-18

**Authors:** Lyuqi Tan, Jilin Tan, Heping Yang, Jieyan Wang, Chunmei Chen, Yanli Peng, Ling Ai, Yurong Tang

**Affiliations:** 1 Salus at Drexel University, Elkins Park, PA 19027, USA; 2 Chongqing Aier Eye Hospital, Aier Eye Hospital Group, Chongqing 400020, P.R. China; 3 Chongqing Aier Nanping Eye Hospital, Aier Eye Hospital Group, Chongqing 400060, P.R. China; 4 Aier Eye Hospital, Jinan University, Guangzhou, Guangdong 510071, P.R. China

**Keywords:** myopia, myopia glasses, scleral ischemia, eyeball movement

## Abstract

The present study examined the effect of wearing myopia glasses on eye movement and scleral blood supply. For this purpose, a total of 30 individuals were recruited for the present self-control study. Under the same fixation distance, the individuals wore 0.00 D and -10.00 D glasses. The amount of eye movement generated when shifting from gazing at a central point to a point light source located at the left or right was measured and compared between the two glasses. The results revealed that the range of eye movement was significantly reduced after wearing -10.00 D glasses. When gazing at the right point light source from the central point, the difference between the rotation distances of the right eye when wearing the 0.00 D glasses and the -10.0 D glasses was 0.73±0.45 mm (t=8.93, P<0.01) and that of the left eye was 0.73±0.43 mm (t=9.34, P<0.01). Similar results were obtained when the left point light source was viewed from a shift in gaze from the central point. On the whole, the present study demonstrates that wearing concave lenses limits eyeball movement. Restricted eyeball movement can affect vascular changes within the extraocular muscles and blood flow, thereby affecting the blood supply to the anterior segment and sclera of the eye, potentially accelerating the development of myopia.

## Introduction

The effects of exercise on the diameter of blood vessels and blood flow within skeletal muscles are profound (1-3); however, it has only recently garnered sufficient attention in the medical community (4). Wearing myopia glasses is a common method to correct myopia. Myopia glasses are concave lenses, which can cause peripheral hyperopic defocus on the retina (5,6). Peripheral hyperopic defocus accelerates the progression of myopia (7,8). Therefore, various myopia glasses have been introduced clinically to correct peripheral hyperopic defocus and prevent the progression of myopia (9,10). However, their effectiveness is limited (11,12).

It would be of interest to determine the reasons for this limitation. Apart from inducing peripheral hyperopic defocus on the retina, it is worthy to examine whether myopia glasses have other effects on the eyes. From an optical theory perspective, it would be prudent to determine whether wearing myopia glasses can also affect eyeball movement, and to determine such an effect in an actual situation. In the case that eyeball movement is affected, the effect this has on the eyeballs should be examined. Any measures that need to be taken in response to this should also perhaps be determined. For this purpose, the present study was designed in an aim to shed some light on the aforementioned questions.

## Subjects and methods

### General information of the study subjects

In a self-control study, 30 subjects from a 150-person hospital team were recruited to participate in an experiment to test eye movement. A total of 7 male and 23 female subjects, aged 18-55 years, were each instructed to wear both 0.00 D and -10.00 D glasses during the experiment. Under both conditions and with the same fixation distance, the amount of movement of the eyes when shifting gaze from a central point to a point light source on the left or right was measured, and the difference between the measurements obtained with the two glasses was compared. The present study followed the principles of the Declaration of Helsinki, in which all subjects signed a consent form when informed of the objectives, risks and benefits of the study. Ethical approval was obtained from the Institutional Review Boards at the Ethics Committee of Chongqing Aier Eye Hospital (Chongqing, China; identifier, no. 202215).

The inclusion criteria were as follows: The corrected visual acuity of both eyes was normal, the dioptre of both eyes was 0-3.00 D, and the age was 18-55 years. The exclusion criteria were as follows: Patients with strabismus or a history of eye surgery.

**Figure 1:**
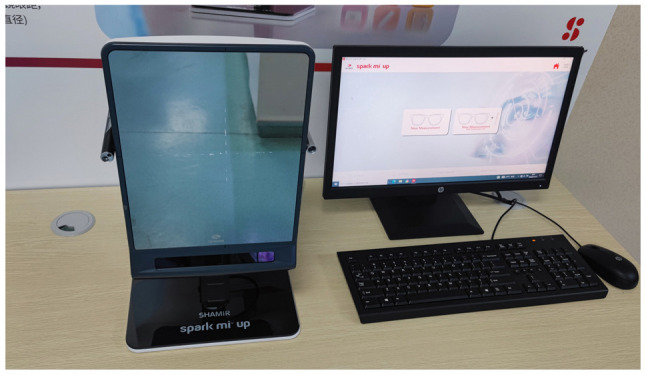
Spark Mi Up pupil distance measuring instrument.

Inspection equipment included the Spark Mi Up pupil distance measuring instrument (Shamir Optical Industry Ltd.) (Fig. 1); a slit lamp holder and a point light source; frames with different pupil distances; two -10.00 D concave lenses; and two 0.00 D glasses.

### Examination principle

The Spark Mi Up pupillary distance measuring instrument captures the central reflection points on the cornea and measures their distance from the nasal midline to obtain the monocular pupillary distance for each eye.

The Spark Mi Up pupillary distance measuring instrument obtains images of the reflected light points of the cornea to calculate the pupillary distance. When the eyeball rotates to the left and right, the movement range of the eyeball can be calculated by measuring the monocular pupillary distance change. The calculation formula is as follows: The rotation amount of the eyeball (mm)=the monocular pupillary distance except for the central gaze point-the monocular pupillary distance at the central gaze point.

### Experimental procedures

**Figure 2:**
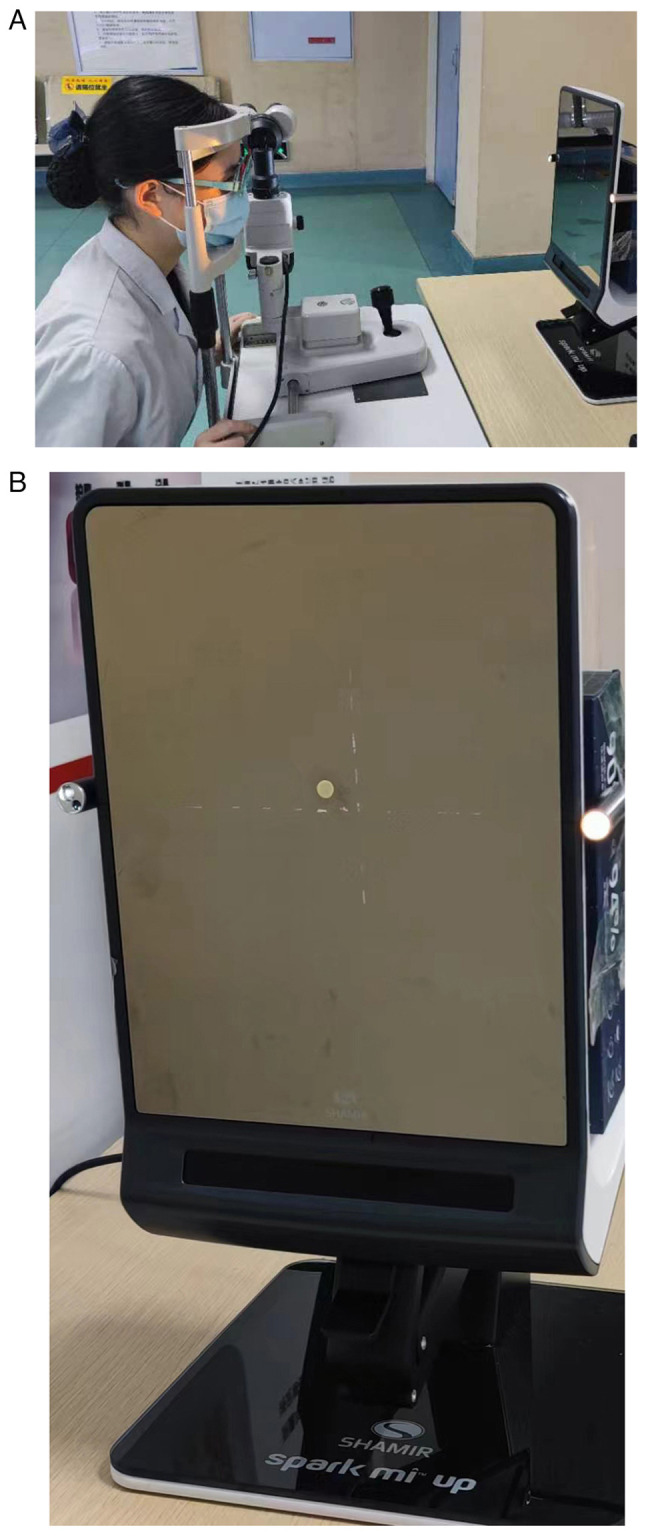
(A) Measuring changes in a single eye's pupillary distance when the subject looked at different targets wearing plain glasses and -10.00 D glasses. (B) Measuring changes in a single eye's pupillary distance when the subject looked at different targets wearing plain glasses and -10.00 D glasses.

The ophthalmic slit lamp holder was fixed 50 cm in front of the Spark Mi Up pupil distance measuring instrument (magic mirror). The subjects were instructed to sit directly in front of the display. Their lower jaw was placed on the lower jaw support with the frontal part leaning against the frontal support. The height of the support was adjusted to ensure that the subjects were seated upright, the head position did not deviate, and the height of the eyes was the same as that of the central point of the pupil distance metre. The point light sources were fixed at the centres of two sides of the screen of the measuring instrument. Ensuring that the heads of the participants could not move during the test, the participants were allowed to first gaze at the central point of the display screen and then at the point light sources on either of the two sides of the display screen. The changes in the amount of eyeball movement from gazing at the central point to gazing at the left or right point light were recorded for the 0.00 D glass and -10.00 D glasses conditions (Fig. 2).

### Statistical analysis

SPSS 19.0 statistical software (SPSS Inc.) was used for statistical analysis. The measurement data were subjected to a Shapiro-Wilk normality test' and confirmed to conform to the normal distribution, and are expressed as the mean ± SD. The differences were tested by paired sample t-tests. A value of P<0.05 was considered to indicate a statistically significant difference.

## Results

After wearing the concave lens, the range of eye movement was markedly reduced, and this difference was significant (Table I). All 30 subjects were first requested to gaze at the right point light source from the central fixation point. The difference between the rotation distance of the right eye when wearing the 0.00 D glass and that wearing the -10.0 D concave lens was 0.73±0.45 mm (t=8.93, P<0.01). The difference between the rotations of the left eye with the two glasses was 0.73±0.43 mm (t=9.34, P<0.01). Both differences were statistically significant.

When the subjects were requested to view the left point light source from the central fixation point, the difference between the rotation distance of the left eye when wearing the 0.00 D glasses and that when wearing the -10.0 D concave lenses was 0.96±0.52 mm (t=10.07, P<0.01). The difference between the rotations of the right eye with the two glasses was 0.80±0.45 mm (t=9.80, P<0.01).

## Discussion

### Wearing myopic glasses limits eyeball movement

In the present study, the subjects were human. The results revealed that apart from hyperopic defocus, the amount of eyeball movement was significantly reduced after wearing the concave lens. When the subjects wore the -10.0 D concave lenses, compared with wearing the 0.00 D glasses, the eyeball movement amount from the point of viewing at the front central gaze to the target of the peripheral visual field was significantly reduced when looking left or right, and the difference was statistically significant. Thus, the hypothesis that hyperopic defocus causes myopia cannot exclude the factor of decreased eyeball movement.

**Figure 3:**
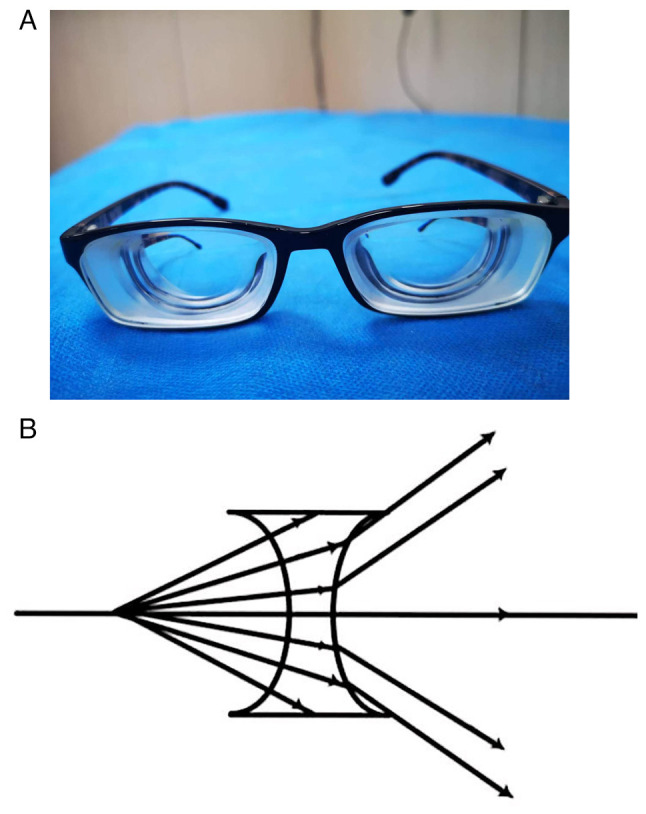
(A) High myopia glasses. (B) Imaging principle of concave lens.

The difference can be explained by the optical principle: When wearing high-dioptre myopia glasses (e.g., -10.0 D) (Fig. 3A), as the concave lens spreads out the light from the macular centre when it passes through the lens (Fig. 3B), the eyeball can see the peripheral visual field without large movement; that is, after wearing the -10.0 D concave lens, the movement amount of the eyeball is decreased when looking at the same external visual field. When a larger visual field is required, as the light reaches the edge of the concave lens and the lens frame, the eyeball movement has to be replaced by the deflection of the head, which further reduces the amount of movement of the eyeball.

When wearing myopia glasses, the frequency of eyeball movement per day remains unaltered; however, the amplitude of each eye movement and the intensity of extraocular muscle movement are significantly reduced. This phenomenon has not previously attracted notable attention, at least to the best of our knowledge. It would thus be of interest to determine its effect on the eyes.

### Restricted eye movement can reduce the blood supply to the sclera and anterior chamber

**Figure 4:**
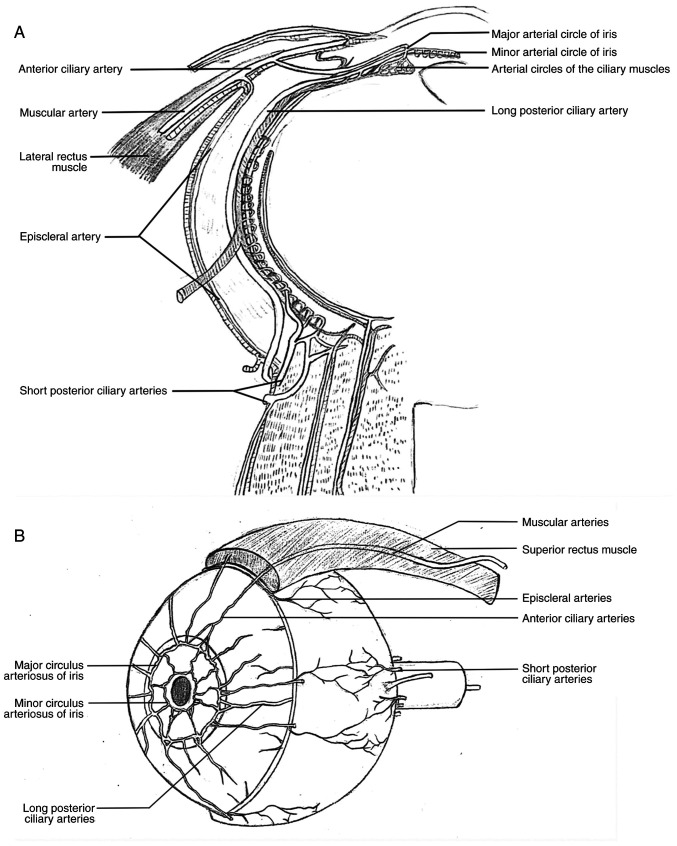
(A) Diagram depicting blood supply to the sclera, ciliary body and iris (sectional view). (B) Diagram depicting blood supply to the sclera, ciliary body and iris (anteromedial view).

Exercise has profound effects on the human vascular system (1-3). McIntosh *et al* (4) reported that the effect of exercise on the diameter and blood flow of arteries within skeletal muscles has been significantly underestimated in the past, only receiving attention recently. There is evidence to suggest that even single sessions of moderate-intensity exercise can increase blood flow velocity within arteries and affect their diameter. Consistent exercise over a period of weeks to months can improve basal blood flow and arterial diameter within skeletal muscles. Similar to other skeletal muscles, the restriction of eye movement inevitably alters the blood flow, luminal shear stress, arterial pressure and tangential wall stress within the extraocular muscles, leading to a reduction in arterial diameter and changes in vascular dilation function, ultimately reducing blood flow speed (13-16). Eye movements in humans are controlled by six extraocular muscles: The superior rectus, inferior rectus, medial rectus, lateral rectus, inferior oblique and superior oblique muscles. The prolonged restriction of eye movement inevitably leads to the narrowing of ocular muscular arteries and a decrease in blood flow. The terminals of the muscular artery are the episcleral arteries and the anterior ciliary arteries. The episcleral arteries are formed by the branches of multiple muscular arteries and the short posterior ciliary arteries (Fig. 4A), with the exception that the external rectus muscle has only one muscular artery, and the other three rectus muscles have two muscular arteries. The effects of changes in the diameter and blood flow of muscular arteries, due to the abundance of these arteries, on the blood supply to the sclera cannot be overlooked. The anterior ciliary arteries are the continuation of the muscular arteries of the rectus muscles. The episcleral arteries are responsible for the blood supply of the sclera. The anterior ciliary arteries participate in the blood supply of the ciliary body and iris (Fig. 4B).

Therefore, limited eye movement not only affects the blood supply to the sclera, but also affects the blood supply to the ciliary body and iris. The occurrence and development of myopia are closely related to scleral ischemia, and the remodeling of the sclera and elongation of the eye axis due to scleral ischemia and hypoxia are recognized pathological processes in the development of myopia (17-21). However, the exact cause of ischemia remains unclear. Nevertheless, this suggests that any factors exacerbating scleral ischemia should be avoided, and any factors improving scleral blood supply should be emphasized. Additionally, the occurrence and development of myopia are associated with accommodative lag, as extensively evidenced in the literature (22-25). Ischemia of the ciliary body and iris will undoubtedly affect the normal functioning of eye regulation. Therefore, wearing myopic glasses, reducing eye movement, will decrease scleral blood supply and affect eye regulation, which is a high-risk factor for accelerating the development of myopia.

For adolescents, the most common behaviors that restrict eye movement, aside from wearing myopia glasses, are likely to include reading at close distances, doing homework and staring at a blackboard or screen, while the behaviors that increase eye movement are outdoor exercise. The occurrence and development of juvenile myopia have a clear association with the time spent participating in outdoor activities. Long-term close reading can lead to the development of myopia, and increasing the time spent participating in outdoor activities can reduce the incidence of myopia (26-30). This is a recognized phenomenon. The reason has always been unclear; however, it cannot be ruled out that it is related to changes in eye movement. The eye movement amplitude is guided by the target seen. The target seen in outdoor activities is not fixed. The wider the field of view, the more rapid the target transformation, and the greater the eye movement amplitude and frequency. In the classroom, during long-term close reading, such as reading a book, looking at a computer, or looking the teaching screen, one can see a tiny field of view, and the gaze target is relatively fixed, which is bound to limit the amplitude and amount of eye movement.

In conclusion, the present study experimentally confirms that wearing myopia glasses not only restricts eye movement, but also allows for the quantitative assessment of the degree of restricted eye movement based on different diopter values. While higher diopter myopia glasses impose more severe movement restrictions and require greater attention, it is crucial to note that any degree of myopia glasses leads to persistent, long-term, cumulative effects on eye movement. The limitations imposed by near-distance reading on eye movement amplitude are notable, with the severity of eye movement restriction directly related to the duration of reading. Therefore, it is recommended that patients wearing myopia glasses and adolescents who unavoidably engage in prolonged near-distance reading, homework, or focus on educational videos should enhance active eye movement or engage in outdoor activities to compensate for restricted eye movement, increase scleral blood supply, and thereby delay or prevent the onset and progression of myopia.

## Figures and Tables

**Table I:** Comparison of eye movement between wearing 0.00 D glasses and wearing -10.0 D lenses.

	Shift from central to right						Shift from central to left					
	OD			OS			OD			OS		
	0.0 D	-10.0 D	Difference	0.0 D	-10.0D	Difference	0·00 D	-10.0 D	Difference	0.0 D	-10.0 D	Difference
Mean (mm)	1.32±0.43	0.58±0.32	0.73±0.45	1.40±0.38	0.67±0.33	0.73±0.43	1.58±0.35	0.78±0.36	0.80±0.45	1.70±0.34	0.74±0.36	0.96±0.52
t-value	8.93			9.34			9.80			10.07		
P-value	<0.01			<0.01			<0.01			<0.01		

## Data Availability

The datasets used and/or analyzed during the current study are available from the corresponding author on reasonable request.
